# Prognostic value of neutrophil gelatinase-associated lipocalin and transpulmonary thermodilution-derived parameters within 48 hours after admission

**DOI:** 10.1186/cc12362

**Published:** 2013-03-19

**Authors:** W Huber, J Rauch, B Saugel, S Mair, M Messer, T Lahmer, C Schultheiss, P Luppa, RM Schmid

**Affiliations:** 1 Klinikum Rechts der Isar, Technical University of Munich, II. Medizinische Klinik, Munich, Germany

## Introduction

Outcome of ICU patients is predicted by multifactorial scores such as APACHE II and SOFA. Furthermore, markers of single organ failure such as the extravascular lung water index (EVLWI) and several biomarkers of acute kidney injury (AKI) have been associated with mortality. Regarding a delayed increase in creatinine, other biomarkers of AKI including neutrophil gelatinase-associated lipocalin (NGAL) have been suggested. It was the aim of our study to compare the predictive capabilities of NGAL and transpulmonary thermodilution (TPTD)-derived parameters within 48 hours after ICU admission.

## Methods

Urinary NGAL, serum creatinine and BUN as well as TPTD-derived parameters were measured 0 hours, 12 hours, 24 hours and 48 hours after ICU admission. Primary endpoint: prediction of ICU mortality. Secondary endpoint: requirement of renal replacement therapy (RRT). Statistics: ROC-AUC. IBM SPSS 20.

## Results

There were 91 patients (34 female, 57 male), 65 ± 14 years, APACHE II score 20.6 ± 8.3, SOFA score 9.3 ± 4.2. Etiology: 10 ARDS, 36 sepsis, 22 cirrhosis, six pancreatitis, eight cardiogenic shock, two CNS-affections, seven various. Mortality was best predicted by SOFA (ROC-AUC: 0.747; *P *0.001) and APACHE II (AUC: 0.705; *P *0.001). Among admission parameters of renal function only NGAL significantly predicted mortality (AUC: 0.647; *P *= 0.023), whereas creatinine (*P *= 0.290) and BUN (*P *= 0.067) were not predictive. ROC-AUCs for NGAL further increased after 12 hours (0.659), 24 hours (0.691; *P *= 0.01) and 48 hours (0.728; *P *= 0.004). NGAL on admission also predicted requirement of RRT during the ICU stay (AUC: 0.678; *P *= 0.015), which was also predicted by creatinine (AUC 0.688; *P *= 0.010) and BUN (AUC: 0.649; *P *= 0.043). Among baseline TPTD parameters, only the pulmonary vascular permeability index (PVPI) significantly predicted mortality (AUC: 0.700; *P *= 0.007). The EVLWI (AUC 0.628; *P *= 0.083) slightly failed significance. Cardiac index, global end-diastolic volume index and heart rate were not predictive. Furthermore, PVPI was the only TPTD-derived parameter to predict requirement of RRT (AUC: 0.693; *P *= 0.009).

## Conclusion

Urinary NGAL on admission significantly predicts mortality, whereas creatinine and BUN were not predictive. The predictive capabilities of NGAL further increased after 12 hours, 24 hours and 48 hours, with the ROC-AUC of NGAL 48 hours exceeding the AUC of the APACHE II score. NGAL on admission also predicts requirement of RRT. Among TPTD-derived parameters, PVPI on admission is significantly associated with mortality and requirement of RRT.

